# Posttraumatic nuchal pseudolipoma in a high school athlete after weight training

**DOI:** 10.1259/bjrcr.20210021

**Published:** 2022-03-09

**Authors:** Conner D Reynolds, Aubrey N. Ingram, Kevin Curley, Joshua Lindsley, Jonas J Kruse, Steven Schultz

**Affiliations:** 1 Texas College of Osteopathic Medicine, University of North Texas Health Science Center, Fort Worth, TX, United States; 2 Transitional Year Residency Program, John Peter Smith Health Network, Fort Worth, TX, United States; 3 Arizona College of Osteopathic Medicine, Midwestern University, Glendale, AZ; 4 Department of Emergency Medicine, University of Texas Southwestern, Dallas, TX, United States; 5 TCU & UNTHSC School of Medicine, Texas Christian University, Fort Worth, TX, United States; 6 Radiology Associates of North Texas, Fort Worth, TX, United States

## Abstract

Pseudolipomas are an uncommon clinical manifestation appearing as a non-encapsulated prominence of subcutaneous fat on MRI. Post-traumatic pseudolipomas (PTLs) are thought to arise from neoadipogenesis following acute or chronic trauma. These are most commonly located on the lower extremities, gluteal, and trochanteric regions. Here, we report a case of PTL in a high school athlete, arising in the posterior neck after weight training with performing barbell squats without neck padding. To our knowledge, this case represents a novel association between PTLs and weight training exercises.

## Case presentation

A 16-year-old male high school athlete presented to the clinic with a progressively enlarging midline posterior neck mass. His symptoms began with “bruising” after a weightlifting session that included heavy barbell squats without neck padding. Over the next several months, this developed into a persistent, progressive, smooth lump without fluctuance, tenderness, or surrounding erythema.

**Figure 1.**
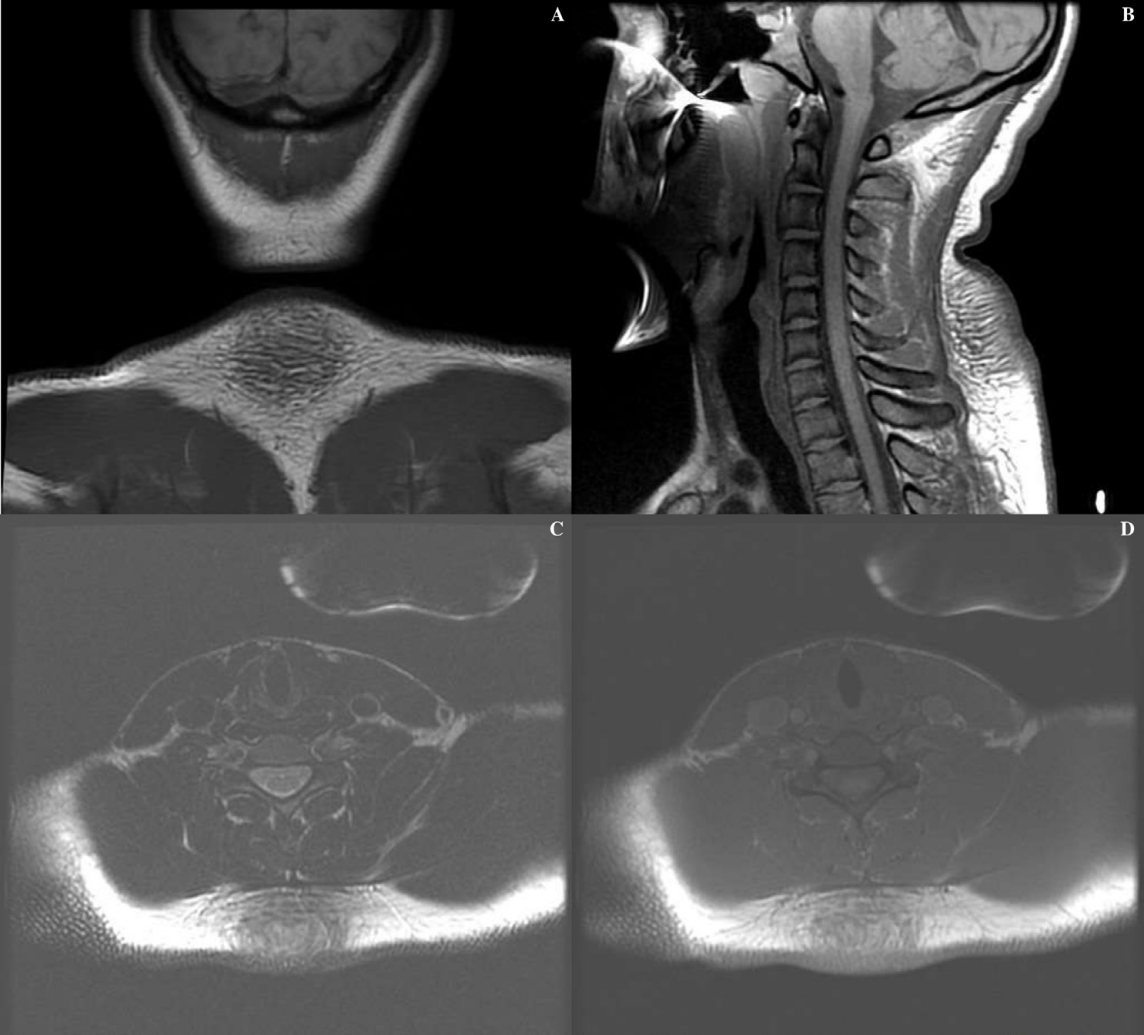
*T*_1_ weighted non-contrasted MRI of the cervical spine at the level of posterior neck mass in (**A**) frontal, (**B**) sagittal, and (**C, D**) coronal planes.

Initial cervical spine X-rays revealed a superficial, ovoid soft tissue density in the posterior neck on the lateral view. Subsequent ultrasonography revealed a 5.7 cm midline, elliptical, echogenic area with skin thickening overlying the area of concern. Further MRI of the cervical spine revealed a midline, unencapsulated, ovoid area of increased subcutaneous fat and thickened, stacked, fibrous septae with overlying dermal thickening (Figure 1A–D), producing a pseudotumor appearance. Taken together, the clinical presentation and radiologic investigations were most consistent with a post-traumatic nuchal pseudolipoma.

## Differential diagnoses

While neck masses have a broad range of differential diagnoses, the overwhelming majority arise in the anterior aspects of the neck.^1^ In fact, a recent single-institution retrospective study and systematic review of posterior neck masses revealed only 19 articles describing 36 patients.^2^ Of these patients, 97% had benign pathologies, including lipomas, nuchal fibromas, schwannomas, epidermal inclusion cysts, lipoblastoma, hemangioma, leiomyoma, lymphangioma, and benign meningioma. One patient (3%) was found to have malignant meningioma.^2^

## Treatment

Treatment modalities for pseudolipoma may include conventional surgical excision or liposuction. Liposuction conveys several advantages over c

onventional surgery for lesions larger than 4 cm, including shorter operating times, reduced risk of intraoperative bleeding, reduced rates of pain, infection, and morbidity, and improved cosmesis.^3,4^ However, surgical excision may be more appropriate for dense, multiseptated appearing lesions on imaging.^4,5^

## Outcome & follow-up

Due to COVID-19 pandemic, the patient is scheduled and waiting to see a dermatologist for further evaluation and management of this condition.

## Discussion

Pseudolipomas are an uncommon clinical manifestation appearing as a non-encapsulated prominence of subcutaneous fat on MRI.^6^ First defined in 1932 as an accumulation of adipose tissue in abnormal locations, post-traumatic pseudolipomas (PTLs) are a poorly defined subgroup of pseudolipomas that seem to arise after either acute severe blunt trauma or chronic repetitive trauma.^4^ PTLs have a female predominance ranging from 3.8 to 12:1. This is possibly explained by a greater proliferative response to estradiol in pre-adipocytes in females compared to males.^4,7^ PTLs are commonly located on the lower extremity as well as the gluteal and trochanteric regions, however there have been cases of PTLs situated on the upper back.^7^ Known colloquially as “tar barreler’s humps,” these chronically induced PTLs are common in a community in Southwest England. Ottery St. Mary is home to a centuries-old annual tradition during which flaming barrels of tar are carried through the streets. In some families, the tradition begins at a young age, and there are stories of several community members with such humps on the back between the shoulders where the barrels rest. Similar to our 16-year-old weightlifter, a recent case was described by Olubaniyi et al of a 32-year-old “tar barreller” whose clinical presentation and imaging findings were consistent with nuchal PTL (*almost identical to our present case*).^8^ The trauma induced by carrying a heavy barrel upon one’s back is comparable to that caused by heavy barbell during squats.

The pathogenesis of PTLs is not well defined, but several postulations have been made. Early theories centered around mechanical and anatomic etiologies such as a traumatic force causing fracture of fat compartments and shearing of anchoring points within Scarpa’s fascia, allowing for protrusion of adipose tissue.^9^ There have been several cases in which no anatomical confirmation could be made, stimulating several new theories. Galea et al proposed that inflammation may be driving neoadipogenesis. Their review referenced studies demonstrating the adipogenic potential of inflammogens using *in vivo* murine models and tissue engineering chambers both with and without fat grafts. Blunt trauma-induced soft tissue inflammation was shown to generate localized elevations in inflammatory chemokines such as interleukin-8 and macrophage inflammatory protein-1β. It was further postulated that the blood matrix from the post-traumatic hematoma and surrounding fibrosis may induce durotactic migration of pre-adipocytes and serve as a nidus for mechanically induced differentiation and proliferation of adipocytes.^4^

The course of development of PTLs is not well defined. The time from trauma to presentation with a lesion ranges from 6 months to 5 years, with a mean between 1 and 2 years.^4,7^ There is an average delay in presentation of 6 months in males compared to females.^4^ There has been no significant data collected that details the time from trauma to the onset of the pseudolipoma, however they have been described to be present upon resolution of the preceding post-traumatic hematoma.^7^

To our knowledge, this case represents a hitherto undocumented association of PTLs with weightlifting exercises. PTLs have an unpredictable course and presentation and are thus poorly recognized by clinicians. It is crucial to elicit a thorough history when working-up lipomatous lesions and to identify any possible cause of acute or chronic trauma, including activities such as weightlifting. Having a benign course and simple, definitive treatment, a swift diagnosis of a PTL can help assuage patient concern and anxiety.

## Learning points

1. PTLs are an uncommon clinical manifestation appearing as a non-encapsulated prominence of subcutaneous fat on MRI, following severe acute or chronic repetitive trauma.
2. Practitioners who encounter young athletic patients with posterior neck masses, in the absence of malignant features, should evaluate their weight training history, particularly concerning barbell squats performed without padding.
3. PTLs have a predilection for females and occur primarily in the lower extremities, gluteal, and trochanteric regions.
